# C14orf166 is a high-risk biomarker for bladder cancer and promotes bladder cancer cell proliferation

**DOI:** 10.1186/s12967-016-0801-4

**Published:** 2016-02-23

**Authors:** Mingkun Chen, Yunlin Ye, Baojia Zou, Suping Guo, Fangjian Zhou, Keshi Lu, Jianye Liu, Zhenzhou Xu, Hui Han, Zhuowei Liu, Yonghong Li, Kai Yao, Cundong Liu, Zike Qin

**Affiliations:** State Key Laboratory of Oncology in South China, 510060 Guangzhou, Guangdong People’s Republic of China; Department of Urology, Cancer Center, Sun Yat-sen University, 510060 Guangzhou, Guangdong People’s Republic of China; Department of Urology, The Third Affiliated Hospital of Southern Medical University, 510630 Guangzhou, Guangdong People’s Republic of China; Department of Urology, Shenzhen Children’s Hospital, 518026 Shenzhen, Guangdong People’s Republic of China; Department of Urology, The Third Xiangya Hospital of Central South University, 410000 Changhsa, Hunan People’s Republic of China; Department of Urology, Hunan Cancer Hospital, 410000 Changsha, Hunan People’s Republic of China; Department of Radiotherapy, Cancer Center, Sun Yat-sen University, 510060 Guangzhou, Guangdong People’s Republic of China

**Keywords:** Bladder cancer, C14orf166, Prognosis, Cell proliferation, G1/S phase

## Abstract

**Background:**

C14orf166 (chromosome 14 open reading frame 166) plays a crucial role in some tumors, but its role in bladder cancer hasn’t been explored.

**Method:**

We determined C14orf166 expression in uroepithelial cell, bladder cancer cells, normal bladder tissues and bladder cancer tissues using quantitative RT-PCR and western blot, we then analyzed the correlation between C14orf166 expression and clinicopathologic characteristics in a cohort of 149 patients with bladder cancer. Finally we downregulated C14orf166 and determined its role in the proliferation of bladder cancer cell lines using MTT assay, colony formation assay and cell cycle assay.

**Results:**

We demonstrated C14orf166 was upregulated in bladder cancer cells and tissues, C14orf166 expression was significantly correlated with larger tumor size (*P* = 0.001), lymph node involvement (*P* < 0.001), histological differentiation (*P* < 0.001), survival time and vital states, and high C14orf166 expression correlated with poor survival, these results suggested C14orf166 served as a high-risk marker for bladder cancer. Knockdown of C14orf166 decreased the proliferation rate and colony formation ability of bladder cancer cells, and arrested cell cycle in G1/S transition. Further analysis showed that C14orf166 knockdown caused abnormal expression of key proteins for G1/S transition, such as Cyclin D1, P21, P27 and Rb phosphorylation.

**Conclusions:**

This study demonstrates that C14orf166 promotes bladder cancer cell proliferation and can be a novel prognostic biomarker for patients with bladder cancer.

## Background

Bladder cancer is a common genitourinary tumor worldwide [1]. It is classified into two types with distinct molecular characteristics and clinical outcomes according to the tumor-node-metastasis (TNM) system. Non-muscle-invasive bladder cancer (stage Ta, carcinoma in situ) accounts for 60 % of bladder cancer diagnoses, and morbidity is very high (50–70 %), its ability to metastasize is very low; the 5-year survival rate is very high, i.e., up to 90 %. Muscle-invasive bladder cancers (≥stage T1) have high ability to metastasize, and the 5-year survival rate is <50 % [2, 3]. To date, it has been reported that some oncogenes play crucial role in the progression of bladder cancer, such as fibroblast growth factor receptor 3 (*FGFR3*) [4, 5], DEP domain–containing 1 (*DEPDC1*) [6], M-phase phosphoprotein 1 (*MPHOSPH1*) [7], and *P73* [8]. Chemotherapy drugs have also been developed for bladder cancer therapy, such as methotrexate, vinblastine, doxorubicin, and cisplatin [9, 10]. However, survival in malignant bladder cancer is still low, and the therapy of bladder cancer remains a challenge. Identifying new genes that promote or suppress bladder cancer development will benefit for bladder cancer therapy.

C14orf166 (is also known CLE or CGI-99), which interacts with the PA subunit of the influenza virus polymerase complex [11], is essential in regulation of viral polymerase activity, viral RNA transcription and replication, and viral particle production [12]. Proteomics analysis has found that C14orf166 interacts with a hepatitis C virus core protein (HCVc), HCVc174, suggesting that C14orf166 modulates replication and function of HCV [13]. Apart from its role in regulation of RNA polymerase activity, C14orf166 promotes development in some tumors. A comparison of the protein expression profiles between pancreatic cancer clinical samples found that C14orf166 levels were higher in samples with lymph node metastasis (LNM) than in non-LNM samples. This suggests that C14orf166 may promote pancreatic cancer metastasis [14]. Howng and colleagues found C14orf166 is high expression in brain tumors, and it blocked ninein phosphorylation by glycogen synthase kinase-3β (GSK3β) [15]. Recently, C14orf166 is demonstrated to correlate with disease progression and poorer outcome in uterine cervical cancer and nasopharyngeal carcinoma [16, 17]. However, the role of C14orf166 in bladder cancer has not been investigated. JAK2/STAT3 signaling promotes bladder cancer progression [18], Xuting Chen et al. [19] found JH2 domain of JAK2 interact with C14orf166, we thought C14orf166 might regulate the progression of bladder cancer.

Here, we studied the role of C14orf166 in bladder cancer, aiming to identify a new target for bladder cancer therapy. We found that C14orf166 was upregulated in bladder cancer cells and tissues compared with normal bladder cells and tissues. High C14orf166 expression was correlated with poor patient survival, and based on analysis of the clinicopathologic characteristics, C14orf166 expression might be a novel prognostic factor for bladder cancer. Tetrazolium (MTT) and colony formation assays revealed that C14orf166 knockdown suppressed cellular proliferation, further analysis found that C14orf166 was a key regulator of G1/S transition.

## Methods

### Cell lines and cell culture

Bladder cancer cells J82, UM3, RT4, 5637 and T24 were cultured in RPMI 1640 medium (Gibco, Grand Island, NY, USA), supplemented with 10 % fetal bovine serum (Gibco), 2 mM l-glutamine (Gibco), 100 μM non-essential amino acids (NEAA), 50 U/mol penicillin, 50 mg/ml streptomycin. Immortalized human uroepithelial cell, SV-HUC-1, was cultured in RPMI 1640 medium, supplemented with 10 % fetal bovine serum, 50 U/mol penicillin and 50 mg/ml streptomycin. All of cells were purchased from American Type Culture Collection (ATCC), and were maintained in a humidified atmosphere at 37 °C with 5 % CO_2_.

### Human bladder cancer specimens

Paraffin-embedded bladder cancer samples were obtained from a cohort of 149 Chinese patients diagnosed with bladder cancer in 2001–2012 at Cancer Center, Sun Yat-sen University, Guangdong Province, People’s Republic of China. The detailed information was presented in Table 1. Clinical and pathologic factors were determined, age, gender, TNM classification, degree of differentiation, tumor number, vital states, tumor size and Recurrence were included. Six fresh bladder tumor tissues which and adjacent bladder tissues were also collected from the same place. The tissues were frozen and stored in liquid nitrogen until further use. All samples were obtained with informed consent and approved by the Guangdong General Hospital Ethics Committee.

**Table 1:** Clinicopathological characteristics of clinical samples and expression of C14orf166 in Human bladder cancer

Characteristics	Number of cases (%)
Age (years)	
<64	81 (47.1)
≥64	91 (52.9)
Gender	
Male	156 (90.7)
Female	16 (9.3)
T classification	
T_1_	91 (52.9)
T_2_	81 (47.1)
N classification	
N_0_	150 (87.2)
N_1_	22 (12.8)
Recurrence	
No	82 (47.7)
Yes	90 (52.3)
Vital status (at follow-up)	
Alive	67 (39.0)
Dead	105 (61.0)
Expression of C14orf166	
Low expression	87 (50.6)
High expression	85 (49.4)
Number	
≥3	64 (37.2)
<3	108 (62.8)
Histological differentiation	
Well	32 (18.6)
Moderate	54 (31.4)
Poor	86 (50.0)
Tumor size	
≥3 cm	100 (58.1)
<3 cm	72 (41.9)

### Small interfering RNAs (siRNAs), RNA extraction and quantitative RT-PCR

In order to knock-down the expression of C14orf166, two C14orf166 siRNAs and their cognate control siRNAs (Scramble) were synthesized by Guangzhou RiboBio Co (Guangdong, China). 20 nm siRNA were transfected into indicated cells in six plates using Lipofectamine RNAiMax Reagent (Life Technologies) according to the manufacturer’s instruction. Total RNA from cultured cells and surgical fresh bladder tumor tissues and adjacent bladder tissues were extracted using Trizol (Life technologies) according to manufacturer’s instructions. cDNA was synthesized from total RNA using ReverTra Ace qPCR RT Kit (TOYOBO). Relative expressions of different genes were detected using SYBR Green PCR Kit (Roche) with the GAPDH gene as an internal reference. The PCR reactions were run using ABI 7500 Real- time PCR System. The comparative C_t_ method (ΔΔC_t_) was used to calculate the relative expression levels.

### Western blot and immunohistochemistry (IHC)

Total protein was extracted from cells using RIPA buffer (P00013C, Beyotime), protein concentration was measured using the BCA protein assay kit (pierce). 30 ug proteins were loaded. Western blot analysis was performed as described [20]. Primary antibodies against C14orf166 (Abcam, ab188326), CyclinD1, P21, P27, retinoblastoma (Rb) and p-Rb were purchased from Santa Cruz Biotechnology. These antibodies were used at a dilution of 1:1000. An antibody to human α-Tubulin (1:5000, Santa Cruz) was used as a loading control. Immunohistochemistry which was used to determine the expression of C14orf166 in bladder cancer tissues and adjacent bladder tissues was performed according to previous report [21], anti-C14orf166 antibody (1:100, Abcam, ab188326) was used.

### 3-(4,5-Dimethyl-2-thiazolyl)-2,5-diphenyl-2H-tetrazolium bromide (MTT) assay

Cells, seeded on 96-well plates, were stained at indicated time points with 20ul sterile MTT dye (5 mg/ml, Sigma) for 4 h at 37 °C, followed by removal of the culture medium and addition of 150ul of DMSO (Sigma). The absorbance was measured at 570, and 655 nm as the reference wavelength. All experiments were performed in triplicates.

### Colony formation assay

Cells were seeded on 6-well plates at a density of 0.5 × 10^3^ cells per well, and cultured for 10 days. Colonies were fixed with 10 % formaldehyde for 5 min and then stained with 1.0 % crystal violet for 30 s.

### Cell cycle assay

Cell cycle assay was performed according to the standard method described previously. Briefly, cells were collected and washed by cold PBS, and fixed with 70 % ethanol for overnight at 4 °C, then cells were washed using cold PBS for three times, and resuspended in PBS buffer containing 100 U/ml RNase A and 50 ug/ml PI at 37 °C for 30 min. Samples were analyzed using BD FACSCalibur cytometer.

### Statistical analysis

All cellular experiments were done at least three repeated, and results were described as the mean ± standard deviation (STDEV) using SPSS version 10.0 software (SPSS, Chicago, IL. USA). Statistical differences were determined by using ANOVA and student’s *t* test for independent samples. Chi square test was used to determine the differences in the expression of C14orf166 between the two categories of tissues. It was used to calculate the p values to indicate the correction between clinical features and C14orf166 expression in bladder cancer. Relative risks of death associated with C14orf166 expression and other predictive variables, such as the T classification, were estimated by using the univariate and multivariate Cox-regression analysis. Kaplan–Meier Survival analysis and the log-rank test were used to plot overall survival curve. A p value of less than 0.05 was considered statistically significant.

## Results

### C14orf166 expression was upregulated in bladder cancer cell lines

**Fig. 1:**
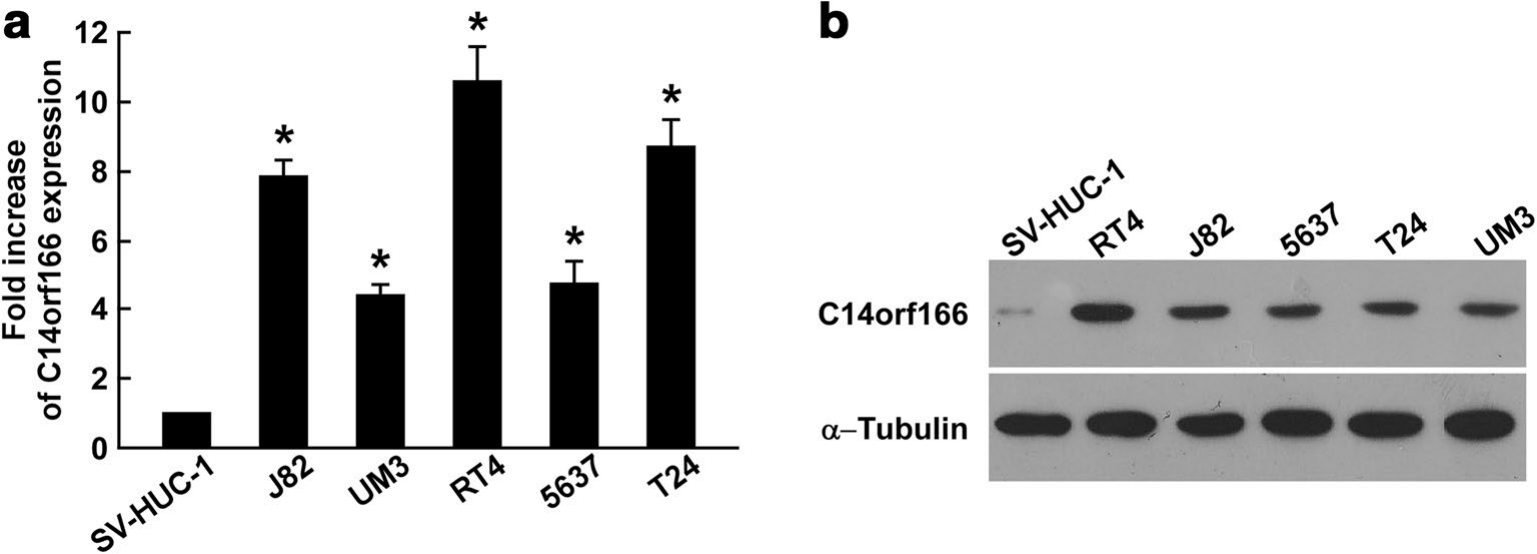
C14orf166 upregulation in human bladder cancer cell lines. **a** Quantitative RT-PCR determination of C14orf166 expression in immortalized human bladder epithelial SV-HUC-1 cells and bladder cancer J82, UM3, RT4, 5637, and T24 cells. Transcription levels were normalized to *GAPDH* expression. **b** Western blot determination of C14orf166 expression in SV-HUC-1, J82, UM3, RT4, 5637, and T24 cells. α-Tubulin was used as the loading control. *P < 0.05

To determine the role of C14orf166 in bladder cancer, we first determined C14orf166 expression in immortalized human bladder epithelial SV-HUC-1 cells and in bladder cancer cell lines. Both quantitative RT-PCR and western blotting revealed that C14orf166 was upregulated in the bladder cancer cell lines (Fig. 1a, b), suggesting that C14orf166 may contribute to the progression of bladder cancer.

### C14orf166 expression was upregulated in bladder cancer tissues

**Fig. 2:**
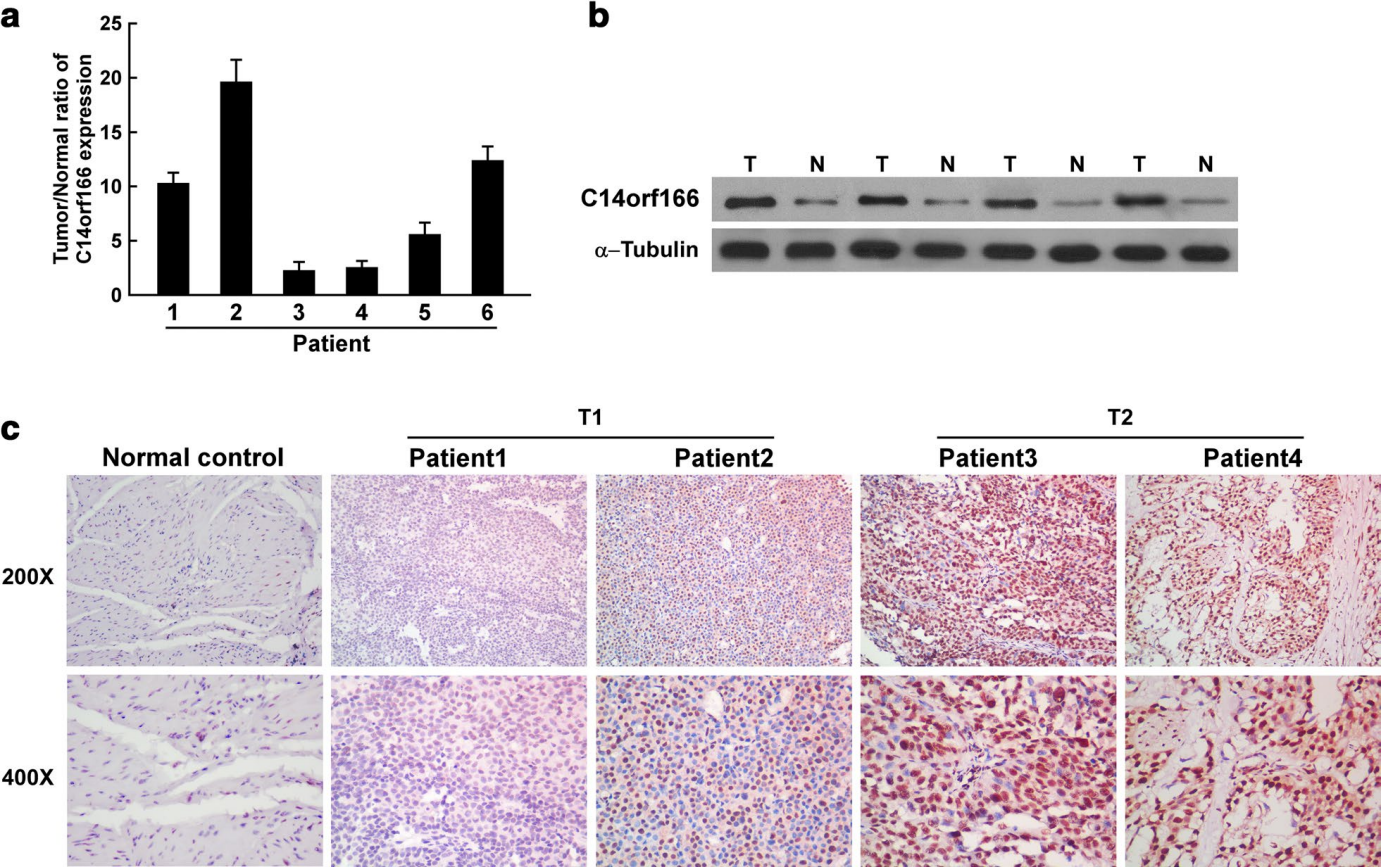
C14orf166 upregulation in bladder cancer tissues. **a** Quantitative RT-PCR determination of C14orf166 expression in bladder cancer tissues and adjacent bladder tissues from six patients. Transcription levels were normalized to *GAPDH* expression. **b** Western blot analysis of C14orf166 expression in bladder cancer tissues and adjacent bladder tissues. α-Tubulin was used as the loading control. **c** IHC determination of C14orf166 expression in different patients with different clinical stages of disease according to T classification (magnification: ×200, ×400)

We also determined C14orf166 expression in clinical samples of bladder cancer. Both quantitative RT-PCR and western blotting revealed that C14orf166 expression was higher in tumor tissues than in normal bladder tissues (Fig. 2a). IHC determined that C14orf166 was upregulated in both T1 and T2 bladder cancer tissues compared to the normal bladder tissues, C14orf166 expression in the T2 samples was higher than that in the T1 samples (Fig. 2b). This indicated that C14orf166 is overexpressed in patients with bladder cancer, suggesting that it may function as a new prognostic biomarker for bladder cancer.

### High C14orf166 expression in primary bladder cancer tissues correlated with poor patient survival

**Fig. 3:**
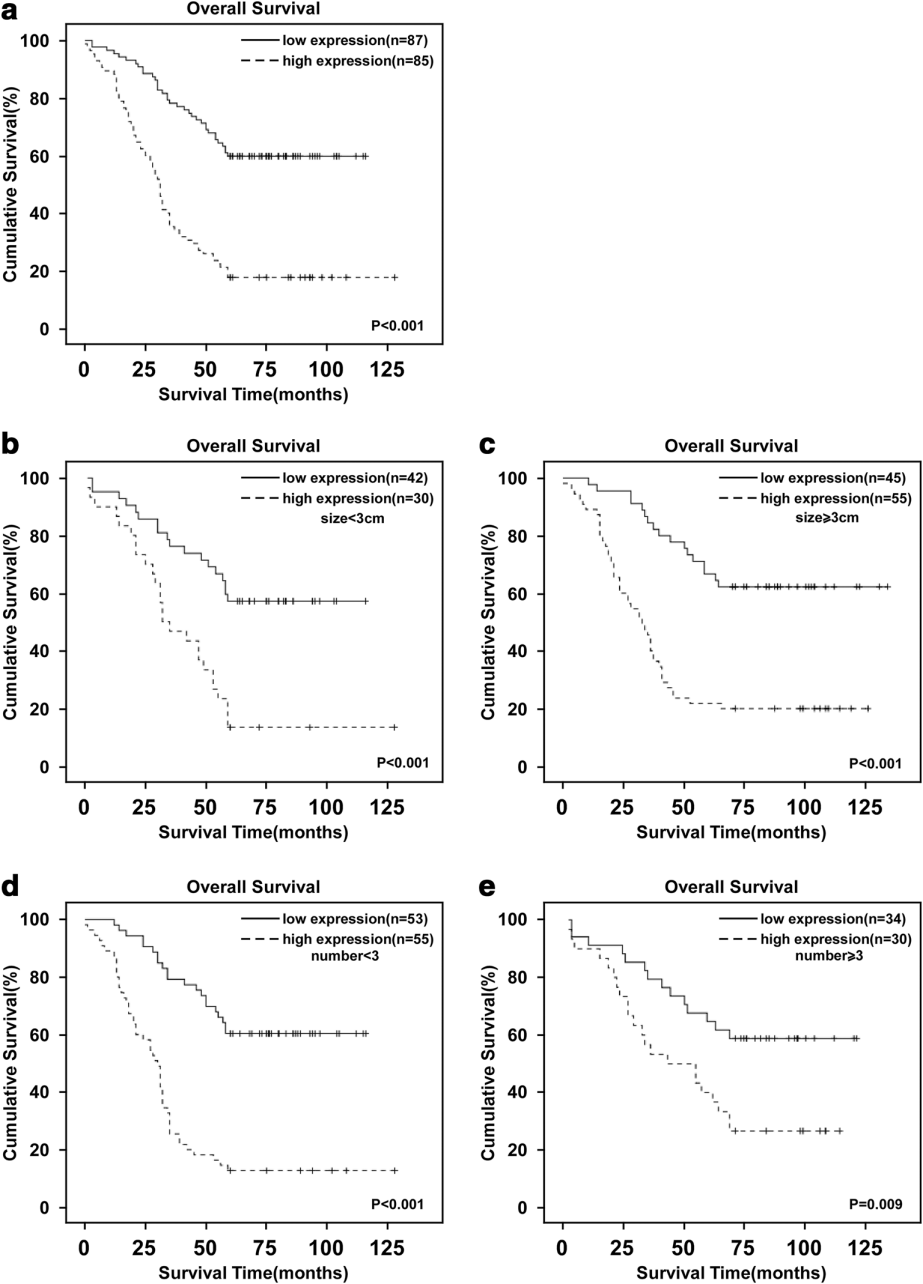
Kaplan–Meier curves with log-rank tests revealed a statistical difference in overall survival. **a** C14orf166 expression and Kaplan–Meier analysis of overall survival (*P* < 0.001). **b** and **c** C14orf166 expression and Kaplan–Meier analysis of overall survival in patients with tumor size <3 cm (*P* < 0.001) (*left*) and with tumor size >3 cm (*P* = 0.008) (*right*), respectively. **d** and **e** C14orf166 expression and Kaplan–Meier analysis of overall survival in patients with tumor number <3 cm (*P* < 0.001) and with tumor number >3 (*P* = 0.001), respectively

To investigate the clinical significance of C14orf166, we determined the relationships between C14orf166 expression and survival in a cohort of 149 patients with bladder cancer (Table 1). Kaplan–Meier survival analysis and the log-rank test determined that the overall survival of patients with high C14orf166 expression was significantly shorter than that of patients with low C14orf166 expression (Fig. 3a). We divided the clinical samples according to the clinicopathologic characteristics, and determined patient survival. Regardless of tumor classification T1 and T2, patients with high C14orf166 expression had significantly shorter survival than those with low C14orf166 expression (Fig. 3b). Regardless of tumor number (n = 3), patients with high C14orf166 expression also had significantly shorter survival than those with low C14orf166 expression (Fig. 3c). These findings suggest that high C14orf166 expression in bladder cancer indicates poor survival.

### Clinical significance of C14orf166 expression in bladder cancer cases

We determined the correlation between C14orf166 expression and the clinicopathologic characteristics of patients with bladder cancer. Tables 2 and 3 show that C14orf166 expression was significantly correlated with larger tumor size (*P* = 0.001), lymph node involvement (*P* < 0.001), histological differentiation (*P* < 0.001), survival time and vital states, which suggested a correlation between higher C14orf166 expression and clinical progression in bladder cancer. However, no significant correlation was found for other clinicopathologic characteristics such as age, sex, recurrence, and tumor number.

**Table 2:** The correlation between C14orf166 expression and clinicopathologic characteristics of bladder cancer patients

Characteristics	Total	PBOV1		Chi square test *P* value
		Low expression	High expression	
Age (years)				
<64	81	41	40	0.993
≥64	91	46	45	
Gender				
Male	156	78	78	0.634
Female	16	9	7	
T classification				
T_1_	91	72	19	<0.001
T_2_	81	15	66	
N classification				
N_0_	150	85	65	<0.001
N_1_	22	2	20	
Recurrence				
Yes	90	39	51	0.046
No	82	48	34	
Histological differentiation				
Well	32	27	5	<0.001
Moderate	54	42	12	
Poor	86	18	68	
Number				
≥3	63	34	30	0.608
<3	108	53	55	
Vital status (at follow-up)				
Alive	67	52	15	<0.001
Dead	105	35	70

**Table 3:** Spearman correlation analysis between C14orf166 and clinical pathologic factors

Variables	C14orf166 expression level	
	Spearman correlation	*P* value
Survival time	−0.413	<0.001
Vital status	0.432	<0.001
T classification	0.605	<0.001
N classification	0.318	<0.001
Histological differentiation	0.551	<0.001
Recurrence	0.152	0.047

We also analyzed the role of C14orf166 in the prognosis of bladder cancer; we defined a relative risk of 1.000 as the baseline in patients with the following characteristics: low C14orf166 expression, T1, N0, and good histological differentiation. We used Cox regression proportional hazard analysis to determine whether C14orf166 could be used as a clinical risk factor. Univariate Cox regression analysis determined that increased C14orf166 expression was associated with significantly increased risk of death (*P* < 0.001), tumor size (*P* = 0.03), lymph node involvement (*P* < 0.001) (Table 4). Multivariate Cox regression analysis found that C14orf166 predicted poor survival when C14orf166 expression, T classification, N classification, and histological differentiation were included in the analysis (*P* < 0.001). Tumor size (*P* = 0.023) and N classification (*P* < 0.001) also predicted unfavorable prognosis. These results suggest that C14orf166 is significantly correlated with the prognosis of bladder cancer, and is an unfavorable prognostic factor for patients with bladder cancer.

**Table 4:** Univariate and multivariate analyses of various prognostic parameters in patients with bladder cancer by Cox-regression analysis

	Univariate analysis			Multivariate analysis		
	No. patients	*P*	Regression coefficient (SE)	*P*	Relative risk	95 % Confidence interval
C14orf166						
Low expression	87	<0.001	1.697 (0.312)	<0.001	5.456	2.962–0.047
High expression	85					
T classification						
T1	91	0.010	−0.584 (0.263)	0.027	0.558	0.333–0.935
T2	81					
N classification						
N_0_	150	<0.001	1.157 (0.285)	<0.001	3.180	1.818–5.564
N_1_	22					
Histological differentiation						
Well	32	0.056	−0.329 (0.154)	0.032	0.720	0.553–0.972
Moderate	54					
Poor	86					

### C14orf166 knockdown inhibited bladder cancer cell proliferation

**Fig. 4:**
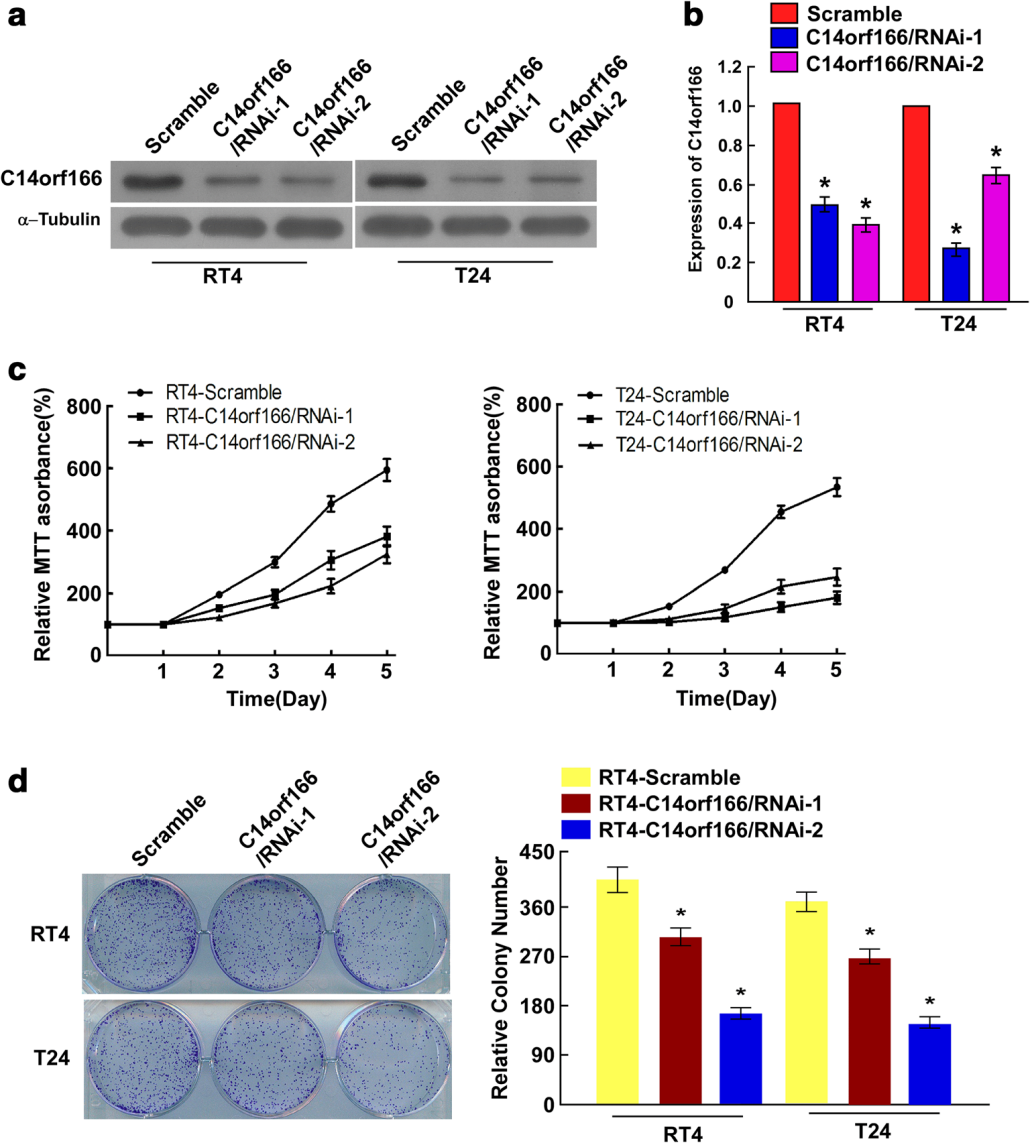
C14orf166 knockdown inhibited the proliferation of bladder cancer cell lines. **a** Western blot determination of the effect of C14orf166 siRNAs in bladder cancer RT4 and T24 cells. **b** Quantitative RT-PCR determination of the effect of C14orf166 siRNAs in bladder cancer RT4 and T24 cells. Transcription levels were normalized to *GAPDH* expression. **c** MTT assay determination of bladder cancer RT4 and T24 cell proliferation following C14orf166 downregulation. **d** Colony formation assay determination of the effect of C14orf166 downregulation on cell proliferation. *P < 0.05

To determine whether C14orf166 regulate cell proliferation, we downregulated its level by siRNAs. Western blotting and quantitative RT-PCR determined that the two siRNAs downregulated C14orf166 expression effectively in the RT4 and T24 bladder cancer cell lines compared to Scramble (Fig. 4a, b).

MTT assay revealed that C14orf166 knockdown reduced the proliferation rate of the two indicated bladder cancer cell lines (Fig. 4c). Colony formation assay determined that downregulating C14orf166 also decreased cell numbers significantly (Fig. 4d). These results suggest that C14orf166 promotes bladder cancer cell proliferation.

### C14orf166 regulated bladder cancer cell proliferation by promoting G1/S transition

**Fig. 5:**
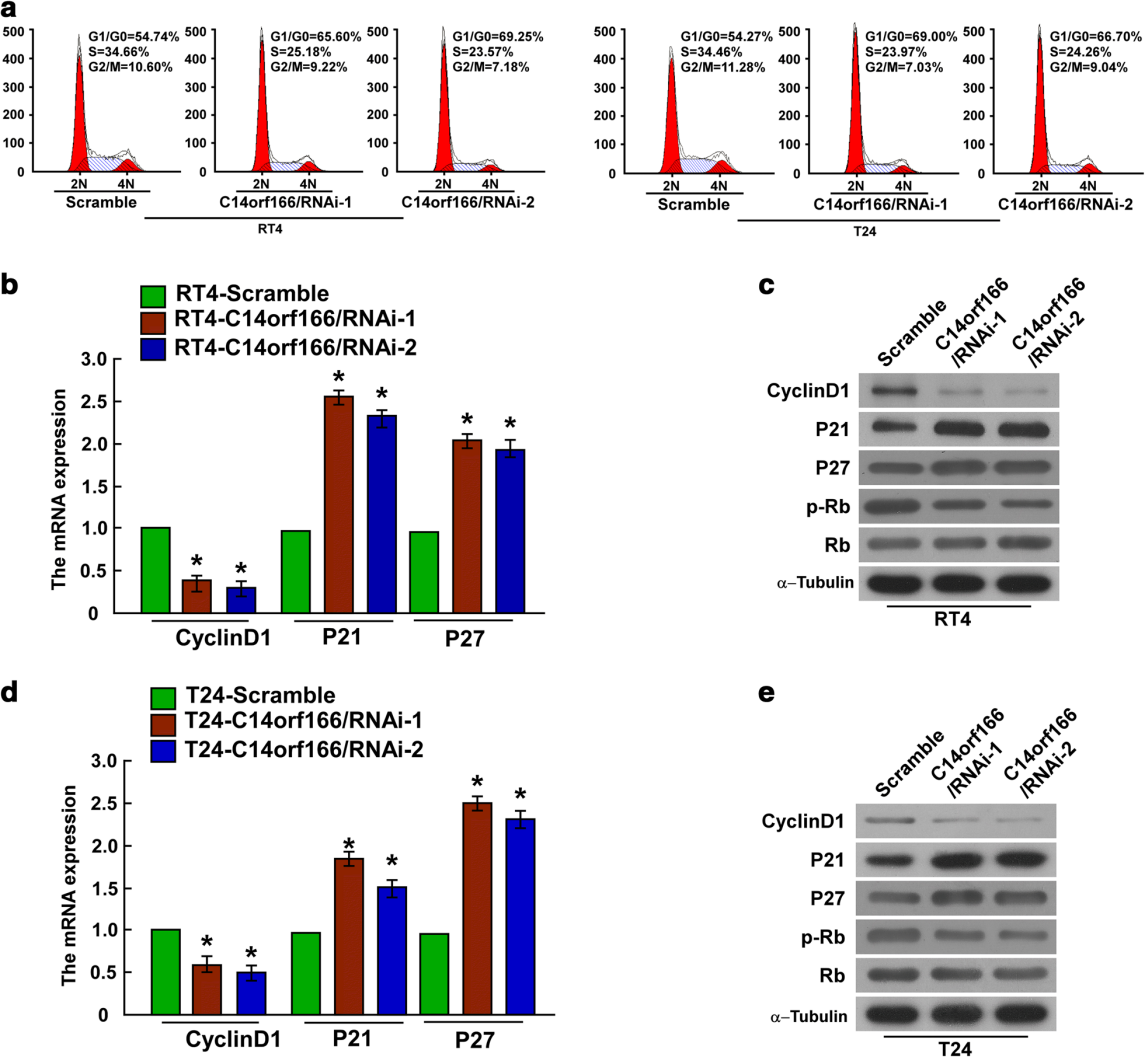
C14orf166 regulated the key proteins that regulate G1/S transition. **a** Cell cycle assay of the effect of C14orf166 knockdown on cell cycle progression. **b** Quantitative RT-PCR determination of Cyclin D1, P21, and P27 expression after C14orf166 knockdown in RT4 cells. Transcription levels were normalized to *GAPDH* expression. **c** Western blot determination of Cyclin D1, p21, p27, Rb, and p-Rb expression in RT4 cells. α-Tubulin was used as the loading control. **d** Quantitative RT-PCR determination of Cyclin D1, P21 and P27 expression following C14orf166 knockdown in T24 cells. Transcription levels were normalized to *GAPDH* expression. **e** Western blot determination of Cyclin D1, p21, p27, Rb, and p-Rb expression in T24 cells. α-Tubulin was used as the loading control. *P < 0.05

We further determined if C14orf166 contributes to cell proliferation through regulating cell cycle proliferation, and found knockdown of C14orf166 increased the population of G1 phase, and decreased the population of S phase (Fig. 5a). We further determined the expression of cell cycle regulatory proteins that control G1/S transition. We downregulated C14orf166 using siRNAs, and both quantitative RT-PCR and western blotting revealed that Cyclin D1 expression was downregulated significantly and P21 and P27 expression was upregulated significantly upon C14orf166 knockdown in RT4 bladder cancer cells. Rb phosphorylation levels were also decreased, but Rb expression was unaltered (Fig. 5b, c). C14orf166 knockdown in T24 cells also downregulated Cyclin D1 significantly and upregulated P21 and P27 significantly; Rb phosphorylation was also reduced (Fig. 5d, e). These results suggest that C14orf166 controls cell proliferation by regulating the proteins that control G1/S transition.

## Discussion

We found that C14orf166 expression is upregulated in bladder cancer cell lines and in primary bladder cancer tissues. C14orf166 promoted cell proliferation by regulating the key regulatory proteins of G1 progression. Disrupted cell cycle control is one of the primary causes of cancer development, where individuals with abnormal expression of the cell cycle control genes have increased risk for bladder cancer [22]. C14orf166 regulates the cell cycle checkpoint proteins, suggesting that it may be a key regulator of bladder cancer progression. We also demonstrated that C14orf166 was associated with clinical characteristics such as tumor size and survival time. High C14orf166 expression predicted unfavorable prognosis and low survival rate. These results suggested that C14orf166 not only functions as an oncogene but also as a novel prognostic biomarker for patients with bladder cancer. This study also demonstrated a new function of C14orf166 in tumor biology.

There are some prognostic biomarkers for bladder cancer, such as tumor protein 53 (*TP53*), *P21*, and tuberous sclerosis 1 (*TSC1*), but these genes only apply to a fraction of bladder cancer patients [23]. Therefore, screening new prognostic biomarkers is essential. We analyzed the relation between C14orf166 and the clinicopathologic parameters of a cohort of 172 clinical samples, and found lower survival rates in patients with high C14orf166 expression compared to patients with low C14orf166 expression. C14orf166 might combine with other clinical biomarkers to help identify patients with higher rates of survival.

C14orf166 is mapped to 14q22.1, and interacts with protein tyrosine phosphatase–interacting protein 51 (PTPIP51) to impact normal mitotic processes and chromosomal division [24]. It also interacts with human ninein (hNinein), a centrosome component, and inhibits hNinein phosphorylation, which suggests that C14orf166 may participate in centrosome architecture and regulate the formation of the centrosome, which plays a vital role in cell cycle progression, thus C14orf166 may regulate cell cycle in this manner [15]. To test the role of C14orf166 in the progression of bladder cancer, we downregulated C14orf166 expression in bladder cancer cells, and found that C14orf166 promoted bladder cancer cell proliferation. We further analyzed the expression of key regulatory proteins of G1/S transition, finding that Cyclin D1, P21, and P27 were downregulated. Cyclin D determines the duration of the G1 phase, and is present in three subtypes: Cyclin D1, Cyclin D2, and Cyclin D3 [25]. Cyclin D1 downregulation arrests cell in G1 phase. Cyclin D1 also binds to cyclin-dependent kinase (CDK) 2, CDK4, and CDK6 to form an active complex that phosphorylates Rb. Rb phosphorylation accelerates G1/S transition [26]. In our study, Rb phosphorylation was decreased. P21 and P27 are cyclin kinase inhibitors, which inhibit cell cycle progression [27, 28]. After C14orf166 knockdown, we found that P21 and P27 expression was upregulated. These findings suggest that C14orf166 promotes cell proliferation by accelerating G1/S transition. However, the role of C14orf166 in apoptosis and cell migration remains to be defined in vitro and in vivo. The in-depth mechanism of C14orf166 in tumorigenesis remains to be elucidated.

## Conclusions

In summary, these data demonstrate that C14orf166 could be a prognostic factor for bladder cancer, and promotes cell proliferation by accelerating G1/S transition.
